# Risk Factors and Adverse Events Poorly Predict Infections and Hypogammaglobulinemia in Granulomatosis with Polyangiitis Patients Receiving Rituximab

**DOI:** 10.1155/2016/8095695

**Published:** 2016-01-18

**Authors:** Emilio Besada

**Affiliations:** Bone and Joint Research Group, Institute of Clinical Medicine, UiT The Arctic University of Norway, 9037 Tromsø, Norway

## Abstract

*Background*. 29 GPA patients from the Northern Norway vasculitis disease registry received rituximab (RTX) induction and maintenance. 24% and 31% had, respectively, severe and chronic infections while 45% had hypogammaglobulinemia and 28% discontinued RTX due to hypogammaglobulinemia. The aim of the study was to examine how known predictors and adverse events interacted with adverse events using structural statistical methods.* Methods*. Five predictors (age, cyclophosphamide, total Ig and CD4/CD8 ratio prior RTX, and type of RTX maintenance regimen) and 4 adverse events (severe and chronic infections, hypogammaglobulinemia, and RTX discontinuation) were modeled in principal component and redundancy analyses.* Results*. The 5 predictors explained 51% of the variance of the GPA cohort. Models including cyclophosphamide exposure and total Ig level predicted best adverse events. However total Ig level has low *R* squared. The 2 best combinations of adverse events explained 13% of the variance of the predictors and adverse events. Only chronic infections were associated with combination of all adverse events (*P* = 0.014). Hypogammaglobulinemia did not seem associated with the other adverse events.* Conclusions*. Traditional risk factors for infections and hypogammaglobulinemia seemed to poorly predict adverse events in our GPA cohort.

## 1. Introduction

Granulomatosis with polyangiitis (GPA) is an ANCA-associated vasculitis (AAV) that affects small- and medium-sized vessels. It frequently involves the upper and lower respiratory tracts and the kidneys. GPA was a uniformly fatal disease prior to the use of cyclophosphamide [1]. The current standard of care of AAV that includes remission induction with high dose of glucocorticoids and cyclophosphamide [2], followed by lower dose of glucocorticoids with either azathioprine or methotrexate for remission maintenance [3, 4], is challenged by rituximab (RTX).

Rituximab is a chimeric monoclonal antibody against CD20 that depletes B-cells. The mechanisms of B-cells depletion include antibody-dependent, cell-mediated, and complement-mediated cytotoxicity with subsequent apoptosis [5]. RTX was effective in randomized controlled trials (RCT) both in induction [6, 7] and in maintenance of remission [8] in AAV and GPA. RTX did not increase the risk of adverse events [6–8] and did not seem to decrease the risk of severe infection during induction compared with CYC [9] in RCT. In 2 retrospective studies, 24% and 41% of GPA patients had severe infections while receiving RTX maintenance [10, 11]. Older age, the cumulative dose of CYC, kidney involvement, low CD4 cell count, and decrease of total Ig after the first 2 g of RTX seemed to increase the risk of severe infection [10]. The risk of hypogammaglobulinemia seemed to be associated with CYC use [12–14] and with the type of RTX maintenance regimen [14] independently of the RTX cumulative dose [13, 14]. However the impact of hypogammaglobulinemia on GPA patients seems to differ in terms of infections and RTX discontinuation between study cohorts [11–14].

In order to predict the risk of adverse events such as infections and hypogammaglobulinemia, we analyzed how patients' characteristics, known predictors, and adverse events interacted with adverse events using structural statistical methods.

## 2. Subjects and Methods

### 2.1. Patients' Characteristics of the Tromsø Cohort at RTX Initiation

29 GPA patients from the Northern Norway vasculitis disease registry received RTX induction and maintenance between April 2004 and 30 September 2011. All patients gave informed written consent at inclusion in accordance with the Declaration of Helsinki. Patients' characteristics are previously published [14]. To summarize, the patients (48% women) with a median age of 50 years at baseline had renal (59%), pulmonary (66%), and orbital-subglottic (62%) involvements. They were 90% ANCA positive (86% PR3-ANCA) at diagnosis. At RTX initiation, patients had median disease duration of 57 months and had received a median total CYC cumulative dose of 17 g (range 0–250).

### 2.2. Treatment Regimen

RTX treatment was initiated as two 1 g infusions 2 weeks apart with coadministration of methylprednisolone 125 mg, paracetamol 1000 mg, and either cetirizine 10 mg or Polaramine 4 mg (rheumatoid arthritis protocol). Due to the observed RTX efficacy and the relapsing nature of GPA, RTX was then readministered preemptively either as a 2 g infusion (1 g twice during a fortnight) annually or as a 1 g infusion biannually (1 g every 6 months).

Patients received a median cumulative RTX dose of 9 g (range 5–13) during a median of 49 months (range 19–88) after RTX initiation. During maintenance, 41% received the regimen of 1 g biannually, 21% received the regimen of 2 g annually, and 38% alternated between regimens.

RTX was added to other immunosuppressive drugs (IDs) (other than prednisolone) in 93% of the patients during a median duration of 24 months (range 1–54). The timing and pace of ID discontinuation were at the discretion of the treating physician, whereas the daily prednisolone dose was tapered from a median of 22.5 mg at baseline and discontinued in a controlled manner. At the last visit, 59% were still treated with long-term preemptive RTX and 90% had discontinued other IDs.

### 2.3. Predictors and Adverse Events Definitions

The 5 chosen predictors of adverse events were age, CYC cumulative dose, total Ig and CD4/CD8 ratio prior RTX, and type of RTX maintenance regimen defined as gram per RTX round (Gpr). These predictors were previously associated with infections and hypogammaglobulinemia [10, 14]. Gpr was calculated as Gpr = (total  cumulative  RTX  dose − 2)/(number  of  rounds − 1). Patients with Gpr = 1 received the regimen of 1 g biannually, patients with Gpr = 2 received the regimen of 2 g annually, and patients with 1 < Gpr < 2 alternated between the two regimens.

Infections were defined either as severe or as chronic. Severe infections included all infections that required either intravenous antibiotics or hospitalization. Chronic infections are symptomatic localized infections lasting over 3 months or more that required multiple antibiotic courses. They are also referred to in the literature as recurrent infections secondary to hypogammaglobulinemia [11].

Hypogammaglobulinemia and hypogammaglobulinemia leading to RTX discontinuation [14] were defined as total Ig level less than 6 g/L during RTX maintenance. The 2 definitions of hypogammaglobulinemia were necessary to include not only patients with severe hypogammaglobulinemia who discontinued RTX maintenance, but also patients with total Ig level under 6 g/L who had either a transitory decrease of Ig or a more benign course [11].

### 2.4. Statistical Analysis

All statistical analysis was done with R (R Project for Statistical Computing, https://www.r-project.org/) with focus on structural methods.

Correspondence analysis (CA) of adverse events during RTX maintenance included all categorical variables: gender, organ involvement, use of concomitant immunosuppressive drugs, type of RTX regimen, age defined in 3 categories (under 40, between 40 and 55, and over 55 years), low and high levels of each immunoglobulin type prior RTX (cut-offs were determined with generalized additive modeling [see Supplementary file and Figure S1 in Supplementary Material available online at http://dx.doi.org/10.1155/2016/8095695]), inverted and normal ratio CD4/CD8 (using cut-off of 1), and low and high cumulative dose of CYC (using median as cut-off).

Principal component analysis (PCA) of the 5 predictors from standardized data determined the variance and significance of each predictor in the Tromsø study cohort. Multivariable analysis with logistic regression using the five predictors individually and in pairs as well as the 2 best PCA dimensions determined the best model explaining the different adverse events. Vectors analysis with biplot after range standardization identified how predictors and adverse outcomes interacted together.

Finally, we determined how the best 2 linear combinations of adverse events explained both predictors and adverse events individually with redundancy analysis (RDA).

## 3. Results

### 3.1. Correspondence Analysis of Adverse Events during RTX Maintenance

Seven patients (24%) had severe infections and 9 (31%) had chronic infections in the Tromsø cohort study, while 8 patients (28%) discontinued RTX due to hypogammaglobulinemia and 13 (45%) had total Ig under 6 g/L during RTX maintenance.

**Figure 1:**
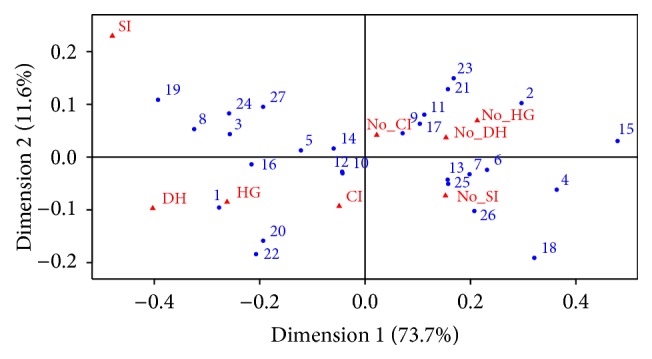
Correspondence analysis of adverse events during RTX maintenance. CI: chronic infection; DH: hypogammaglobulinemia leading to RTX discontinuation; HG: hypogammaglobulinemia with total Ig < 6 g/L; SI: severe infection. No_CI: absence of chronic infection; No_DH: absence of hypogammaglobulinemia leading to RTX discontinuation; No_HG: absence of hypogammaglobulinemia with total Ig < 6 g/L; No_SI: absence of severe infection. 1: men; 2: women; 3: kidney involvement; 4: no kidney involvement; 5: lung involvement; 6: no lung involvement; 7: orbital and subglottic involvement; 8: no orbital and subglottic involvement; 9: use of MTX during RTX; 10: no MTX use; 11: use of AZA during RTX; 12: no use of AZA; 13: use of MMF during RTX; 14: no use of MMF; 15: RTX maintenance with the 2 g annually regimen; 16: other maintenance regimes; 17: age under 40 y at baseline; 18: age between 40 and 55; 19: age over 55 years; 20: low IgG level prior RTX; 21: high IgG prior RTX; 22: low IgA level prior RTX; 23: high IgA prior RTX; 24: inverted CD4/CD8 ratio prior RTX; 25: normal ratio prior RTX; 26: low CYC cumulative dose; 27: high CYC cumulative dose.

In the CA, the presence and absence of adverse events were grouped in the opposite ends of the map, except for chronic infections that remained close to the centroid (Figure 1). The 2 types of hypogammaglobulinemia and severe infection were utterly separated. Being a woman, being younger than 55 years, being without kidney and lung involvement, being with orbital-subglottic involvement, being previously treated with lower CYC cumulative dose, and receiving the RTX regimen of 2 g annually were factors protective against adverse events (Figure 1). On the other hand, being a man, being older than 55 years, being with kidney and without orbital-subglottic involvements, being with ratio CD4/CD8 < 1, being previously treated with higher CYC cumulative dose, and receiving RTX maintenance regimen other than 2 g annually increased the risk of adverse events (Figure 1). Being a man and having lower levels of IgG and IgA prior RTX (resp., lower than 7.5 and 1.6 g/L) seemed to be more closely associated with hypogammaglobulinemia than severe infections (Figure 1).

### 3.2. Principal Component Analysis of the 5 Predictors

**Figure 2:**
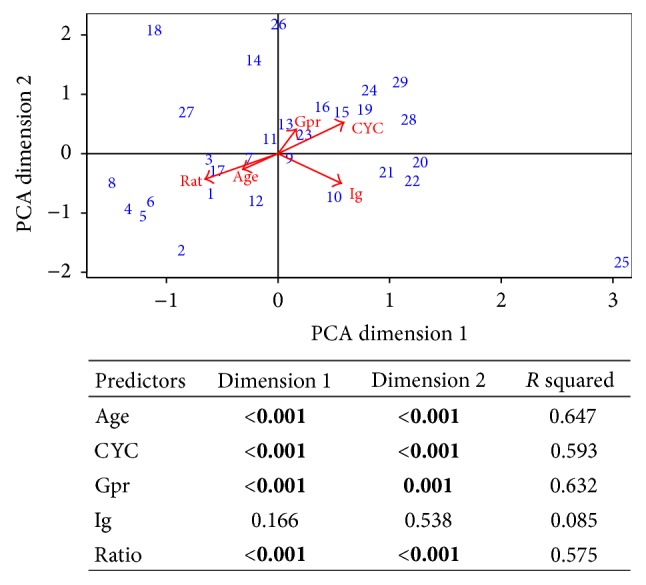
Principal component analysis of the five predictors during RTX maintenance. Data is standardized. *P* values and *R* squared of each predictor are presented in the included table. 1–29: patients' study number; age; CYC: cyclophosphamide cumulative dose; Gpr: gram of RTX per round; Ig: total level of immunoglobulin prior rituximab; Rat: ratio CD4/CD8 prior rituximab.

PCA identified one outlier in the study cohort (Figure 2). The outlier represented an older patient who had received 250 g of CYC prior RTX. The best linear combination of the five predictors explained 51% of the variance of the study cohort. All predictors, except for total Ig levels prior RTX, were significant in the PCA (Figure 2).

### 3.3. Multivariable Analysis of Adverse Events

#### 3.3.1. Severe Infection

The pair of CYC cumulative dose and age was the strongest predictor of severe infection in the study cohort. Being 10 years older or receiving 10 more grams of CYC more than doubled the risk of severe infection although it was only significant for the CYC cumulative dose (*P* = 0.011) (Figure S2 and Table S1).

#### 3.3.2. Chronic Infection

The strongest predictor of chronic infection was the ratio CD4/CD8 prior RTX. Every increase of 0.2 in ratio prior to RTX increased the risk of chronic infection by 14% although this was not found significant (*P* = 0.210) (Table S2).

#### 3.3.3. Hypogammaglobulinemia Defined as Total Ig < 6 g/L

The pair of CYC cumulative dose and total Ig level prior RTX was the strongest predictor of hypogammaglobulinemia in the study cohort. Receiving 10 more grams of CYC and decrease of 1 g/L of total Ig level increased the risk of hypogammaglobulinemia, respectively, by 51% and 67%, although this was only found significant for total Ig level prior RTX (*P* = 0.044) (Figure S3 and Table S3).

#### 3.3.4. Hypogammaglobulinemia Leading to Discontinuation

The strongest predictor in the univariable analysis was the cumulative dose of CYC although it was not significant (*P* = 0.137) (Table S4). The best model predicting discontinuation due to hypogammaglobulinemia included the cumulative dose of CYC and the level of total Ig prior RTX (Figure S4). The risk of discontinuation due to hypogammaglobulinemia increased by 54% for each decrease of 1 g/L of total Ig and by 30% for every 10 g increase of CYC cumulative dose, although none were significant (Table S4).

### 3.4. Analysis of Outcomes Using PCA of the 5 Predictors Using Range Standardization

**Figure 3:**
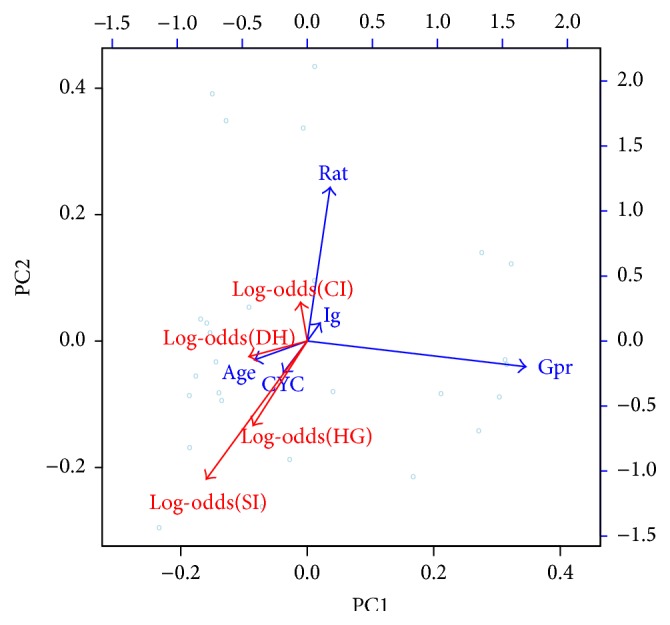
Two-dimensional biplot of adverse events using the principal component analysis of the five predictors. CI: chronic infection; CYC: cyclophosphamide cumulative dose; DH: discontinuation due to hypogammaglobulinemia; Gpr: gram of RTX per round; HG: hypogammaglobulinemia defined as total Ig < 6 g/L; Ig: total level of immunoglobulin prior rituximab; Rat: ratio CD4/CD8 prior rituximab; SI: severe infection.

The vectors of severe infection, hypogammaglobulinemia, and RTX discontinuation due to hypogammaglobulinemia had the same direction in the PCA (Figure 3). The 2 first vectors were almost superposed while the vector of RTX discontinuation due to hypogammaglobulinemia was lying towards the chronic infection vector (Figure 3). Being older, receiving a higher dose of CYC, lower ratio and total Ig level prior RTX, and the regimen of 1 g biannually seemed to increase the risk of severe infection and of hypogammaglobulinemia (Figure 3).

### 3.5. Redundancy Analysis of Predictors and Adverse Events Using the Best Linear Combination of Adverse Events

**Figure 4:**
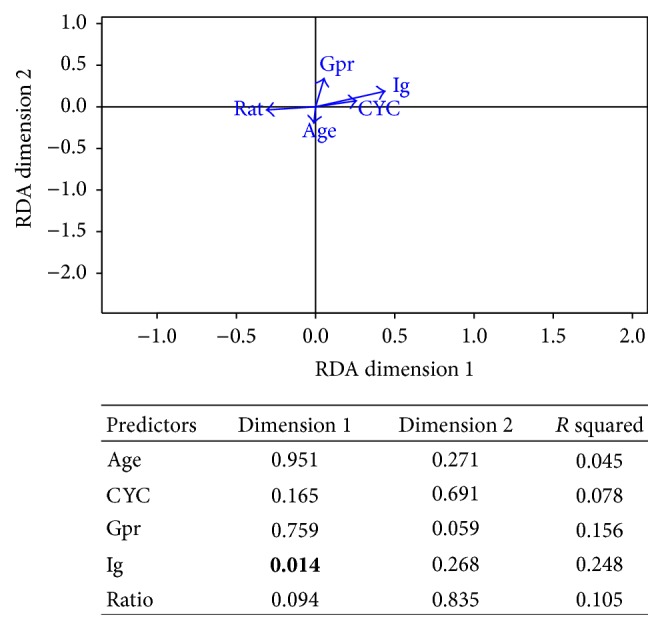
Redundancy analysis of the five predictors using the two best combinations of outcomes. *P* values and *R* squared of each predictor are presented in the included table. Age: age in years at rituximab initiation; CYC: cyclophosphamide cumulative dose; Gpr: gram of RTX per round; Ig: total level of immunoglobulin prior rituximab; Rat: ratio CD4/CD8 prior rituximab; severe I: severe infection.

Severe and chronic infections, hypogammaglobulinemia, and discontinuation due to hypogammaglobulinemia explained together an average of 12.6% of the variance of the 5 predictors (Figure 4). The adverse events only explained significantly the variance of the total Ig prior to RTX (Figure 4). Of interest, the vectors of total CYC cumulative dose and of total Ig level prior RTX had the same direction in the RDA.

**Figure 5:**
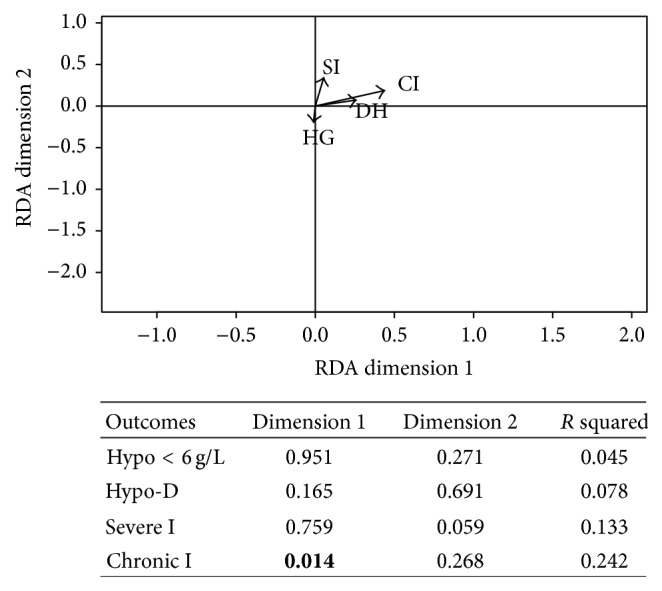
Redundancy analysis of the outcomes using the two best combinations of outcomes. *P* values and *R* squared of each outcome are presented in the included table. CI: chronic infection; DH: discontinuation due to hypogammaglobulinemia; HG: hypogammaglobulinemia defined as total Ig < 6 g/L; SI: severe infection.

The 2 best combinations of adverse events explained an average of 12.5% of the variance of the 4 adverse events (Figure 5). Only the risk of chronic infection was found significant in the RDA. Vector of hypogammaglobulinemia defined as total Ig < 6 g/L had a different direction compared with the other outcomes (Figure 5).

## 4. Discussion

The 5-predictor model explained 51% of the variance of the cohort although this was not significant for total Ig levels prior RTX. CYC and total Ig level prior RTX were important predictors of the different adverse events, apart from chronic infection. However the 2 best combinations of adverse events explained 13% of the variance of both the predictors and the adverse events, only being significant for total Ig level prior RTX and chronic infection, respectively.

Total Ig level prior RTX seemed to be the most important predictor of adverse events during RTX maintenance. However predicting adverse events using total Ig level could be problematic due to the inherent large prediction interval associated with low *R* squared, as observed in our cohort. Total CYC cumulative dose was a significant predictor of severe infections and possibly of hypogammaglobulinemia. It seemed more reliable in detecting patients at risk of adverse events since it had a smaller prediction interval. Nevertheless the total cumulative dose of CYC and the total Ig level prior RTX were closely related in terms of combination of adverse events.

The type of RTX maintenance regimen was not a strong predictor of adverse events; however it is a modifiable factor, like the total cumulative dose of CYC. Categorical variables such as gender and organ involvement were not used in modeling the outcomes and could still be of importance when predicting adverse events during RTX maintenance.

Chronic infections seemed to be more associated with severe infection and discontinuation due to hypogammaglobulinemia than with predictors. The 5 predictors did not manage to model the risk of chronic infection, while chronic infection was significantly associated with adverse events in the outcome analysis.

Hypogammaglobulinemia, discontinuation due to hypogammaglobulinemia, and severe infections seemed closely related in the study cohort whereas hypogammaglobulinemia was not associated with discontinuation of RTX and severe infection in the outcome analysis. Hypogammaglobulinemia defined as low levels of either IgG or total Ig levels could lack specificity when compared with severe infections and discontinuation due to hypogammaglobulinemia, since half of the patients receiving RTX had erratic IgG concentration-time profile in one study [15]. Another possible explanation is the timing of the different adverse events since transitory hypogammaglobulinemia could occur early during RTX treatment [15], while hypogammaglobulinemia leading to RTX discontinuation usually occurred later [14].

In our cohort of GPA patients, outcome was poorly associated with predictors and with individual adverse events making it difficult for physicians to stratify patients' risk individually. To make risk stratification more complicated, some adverse events either lacked specificity or were unrelated to predictors. When physicians are not able to predict adverse events, modifiable factors such as limiting the cumulative dose of CYC and limiting the number of times B-cells are depleted are of importance when treating GPA patients. More studies are clearly needed to clarify not only the timing, but also the dosage of RTX therapy, since lower RTX doses at induction [16, 17] and during maintenance [8] are also effective in AAV/GPA.

## 5. Principal Findings from the Structural Statistical Analysis

The 5 predictors explained 51% of the variance of the GPA cohort from Tromsø.

Total Ig was a strong predictor of adverse events, but it was not reliable due to a large prediction interval.

The best 2 combinations of outcomes explained only 13% of the variance of all 5 predictors and of all 4 adverse events.

Vectors of total cumulative dose of CYC and of total Ig level prior RTX had the same direction in the outcome analysis indicating that the 2 predictors were closely related.

Vectors of discontinuation of RTX due to hypogammaglobulinemia and of severe infection have the same direction in the outcome analysis. These 2 adverse events were closely related, contrary to hypogammaglobulinemia defined as total Ig < 6 g/L.

There were no predictors for chronic infection. Chronic infection is only significantly associated with adverse events.

## Supplementary Material

The Supplementary Material comprises analysis with generalized additive models of the decrease of immunoglobulin types during RTX maintenance. It also includes univariable and multivariable analysis with logistic regression for each adverse event using the 5 predictors and modelling each adverse event using a two-dimensional biplot of the best pairs of predictors.
