# Supplementary material for: Risk Factors and Adverse Events Poorly Predict Infections and Hypogammaglobulinemia in Granulomatosis with Polyangiitis Patients Receiving Rituximab
Source: Autoimmune Dis. 2016 Jan 18;2016:8095695. doi: 10.1155/2016/8095695 (PMC4739481; doi:10.1155/2016/8095695)
Supplement: Supplementary file 1 — The Supplementary Material comprises analysis with generalized additive models of the decrease of immunoglobulin types during RTX maintenance. It also includes univariable and multivariable analysis with logistic regression for each adverse event using the 5 predictors and modelling each adverse event using a two-dimensional biplot of the best pairs of predictors. [file 8095695.f1.pdf]

Supplementary file

Reduction of immunoglobulin types during RTX maintenance in the Tromsø study cohort

Ig levels at nadir during RTX maintenance were partly dependent on the levels of Ig at baseline in linear regression models. (14). This relationship was strong with IgM (R^2=0.561, P < 0.001), to a lesser extent with IgG (R^2 = 0.179, P = 0.013) and not significant with IgA (R^2=0.079 p=0.076) (14). In generalized additive models (GAM), IgA and IgM nadir levels during RTX maintenance were best predicted from baseline using models with respectively 2.36 and 4.07 estimated degrees of freedom (edf) while IgG and total Ig nadir were still best predicted with linear regression models (edf=1) (Figure S1).

Levels of IgA and IgM prior RTX explained respectively 35 and 62 % of their nadir levels under RTX (p=0.004 and p<0.001) in GAM. Levels of IgG and total Ig explained 18 and 19 % of their nadir levels. Of interest many GPA seemed to reach a plateau of IgM at around 0.5 g/L during RTX maintenance and patients with high levels of IgA prior to RTX seemed to have deeper decrease during RTX maintenance (Figure S1).

IgG, IgA, IgM and total Ig levels prior RTX at risk to levels lower than the normal range are respectively 7.5, 1.6, 1.0 and 8.9 g/L.

Table S1

Odds ratio for severe infection during RTX maintenance using the 5 predictors

Univariable analysis with logistic regression

	Intercept	Coefficient	Z (coif)	p-value	OR	AIC
A	-3.46	0.047	1.34	0.182	1.048	33.9
C	-3.38	0.057	2.31	0.021	1.059	26.0
G	2.38	-2.834	-1.55	0.121	0.059	32.3
I	-0.973	-0.016	-0.118	0.906	0.985	36.0
R	1.10	-2.09	-1.75	0.080	0.123	30.6

Analysis of pairs of factors with logistic regression

	Coef1	Coef2	p(1)	p(2)	OR(1)	OR(2)	AIC
A+C	0.092	0.083	0.056	0.011	1.10	1.09	23.1
A+G	0.034	-2.54	0.350	0.175	1.03	0.08	33.3
A+I	0.047	0.002	0.185	0.986	1.05	1.00	35.9
A+R	0.040	-1.80	0.306	0.113	1.04	0.16	31.4
C+G	0.078	-5.37	0.0213	0.152	1.08	0.01	23.4
C+I	0.062	0.091	0.025	0.609	1.06	1.10	27.7
C+R	0.060	-1.62	0.025	0.120	1.06	0.20	24.5
G+I	-2.85	-0.016	0.122	0.912	0.06	0.98	34.3
G+R	-2.83	-1.89	0.146	0.109	0.06	0.15	29.5
I+R	-0.032	-2.11	0.843	0.080	0.97	0.12	32.5
PCA1+PCA2	-3.91	-5.35	0.086	0.101	0.02	0.01	27.3

A: age; C: Cyclophosphamide cumulative dose; G: RTX gram per round; I: total immunoglobulin level prior RTX; R: ratio CD4/CD8 prior RTX

AIC: Akaïke information criterion; Coef: coefficient; OR: odds ratio

Table S2
Odds ratio for chronic infection during RTX maintenance using the 5 predictors

Univariable analysis with logistic regression

	Intercept	Coef	St. coef	p-value	OR	AIC
A	-0.910	0.002	0.090	0.928	1.00	39.9
C	-1.07	0.007	0.834	0.401	1.01	39.1
G	-0.353	-0.333	-0.317	0.751	0.72	39.8
I	-0.493	-0.028	-0.225	0.822	0.97	39.9
R	-1.70	0.654	1.25	0.210	1.92	38.3

Analysis of pairs of factors with logistic regression

	Coef1	Coef2	p1	p2	OR1	OR2	AIC
A+C	0.001	0.007	0.968	0.406	1.00	1.01	41.2
A+G	<0.001	-0.333	1.00	0.761	1.00	0.72	41.8
A+I	0.002	-0.027	0.937	0.825	1.00	0.97	41.9
A+R	0.006	0.663	0.835	0.205	1.01	1.94	40.3
C+G	0.007	-0.181	0.428	0.867	1.01	0.83	41.2
C+I	0.007	-0.022	0.410	0.860	1.01	0.98	41.1
C+R	0.011	0.812	0.241	0.137	1.01	2.25	38.9
G+I	-0.322	-0.026	0.760	0.834	0.72	0.97	41.8
G+R	-0.388	0.660	0.717	0.207	0.68	1.93	40.2
I+R	-0.036	0.665	0.728	0.205	0.96	1.94	40.2
PC1+PC2	-0.269	1.54	0.797	0.272	0.76	4.66	40.6

Table S3
Odds ratio for hypogammaglobulinemia < 6g/L using the 5 predictors

Univariable analysis with logistic regression

	Intercept	Coef	St Coef	p	OR	AIC
A	-1.55	0.028	1.081	0.280	1.03	42.6
C	-1.31	0.035	1.92	0.056	1.04	38.2
G	1.87	-1.57	-1.46	0.143	0.21	41.5
I	4.03	-0.400	-2.03	0.043	0.67	37.1
R	1.11	-1.04	-1.67	0.096	0.35	40.4

Analysis of pairs of factors with logistic regression

	C1	C2	p1	p2	OR1	OR2	AIC
A+C	0.051	0.048	0.128	0.031	1.05	1.05	37.4
A+G	0.019	-1.37	0.472	0.213	1.02	0.25	43.0
A+I	0.027	-0.400	0.317	0.046	1.03	0.67	38.1
A+R	0.025	-0.973	0.367	0.111	1.03	0.38	41.5
C+G	0.037	-1.56	0.058	0.191	1.04	0.21	38.2
C+I	0.041	-0.519	0.068	0.044	1.04	0.60	33.1
C+R	0.037	-1.02	0.058	0.133	1.04	0.36	37.4
G+I	-1.78	-0.410	0.146	0.040	0.17	0.66	36.7
G+R	-1.57	-0.992	0.162	0.101	0.21	0.37	40.2
I+R	-0.522	-1.54	0.020	0.043	0.59	0.21	33.7
PC1+PC2	-2.11	-3.29	0.071	0.072	0.12	0.04	37.8

Table S4

Odds ratio for discontinuation due to hypogammaglobulinemia using the 5 predictors

Univariable analysis using logistic regression

	Intercept	Coef	St Coef	p-value	OR	AIC
A	-1.40	0.009	0.328	0.743	1.01	38.1
C	-1.93	0.026	1.49	0.137	1.03	33.6
G	1.78	-2.15	-1.48	0.140	0.12	35.3
I	-2.46	-0.332	-1.56	0.118	0.72	34.5
R	-0.703	-0.202	-0.358	0.721	0.82	38.0

Analysis of pairs of factors with logistic regression

	C1	C2	p1	p2	OR1	OR2	AIC
A+C	0.013	0.028	0.684	0.137	1.01	1.03	35.5
A+G	-0.004	-2.20	0.895	0.142	1.00	0.11	37.3
A+I	0.007	-0.331	0.802	0.121	1.01	0.72	36.4
A+R	0.008	-0.182	0.773	0.747	1.01	0.83	39.9
C+G	0.028	-2.16	0.150	0.192	1.03	0.11	33.4
C+I	0.026	-0.433	0.139	0.110	1.03	0.65	31.6
C+R	0.026	0.054	0.136	0.927	1.03	1.06	35.6
G+I	-2.58	-0.357	0.143	0.108	0.08	0.70	33.4
G+R	-2.14	-0.157	0.142	0.777	0.12	0.85	37.2
I+R	-0.351	-0.327	0.111	0.599	0.70	0.72	36.2
PC1+PC2	-2.29	-0.611	0.117	0.690	0.10	0.54	36.6

Figure S1

Generalized additive models predicting nadir level from the baseline of each immunoglobulin type during rituximab maintenance.

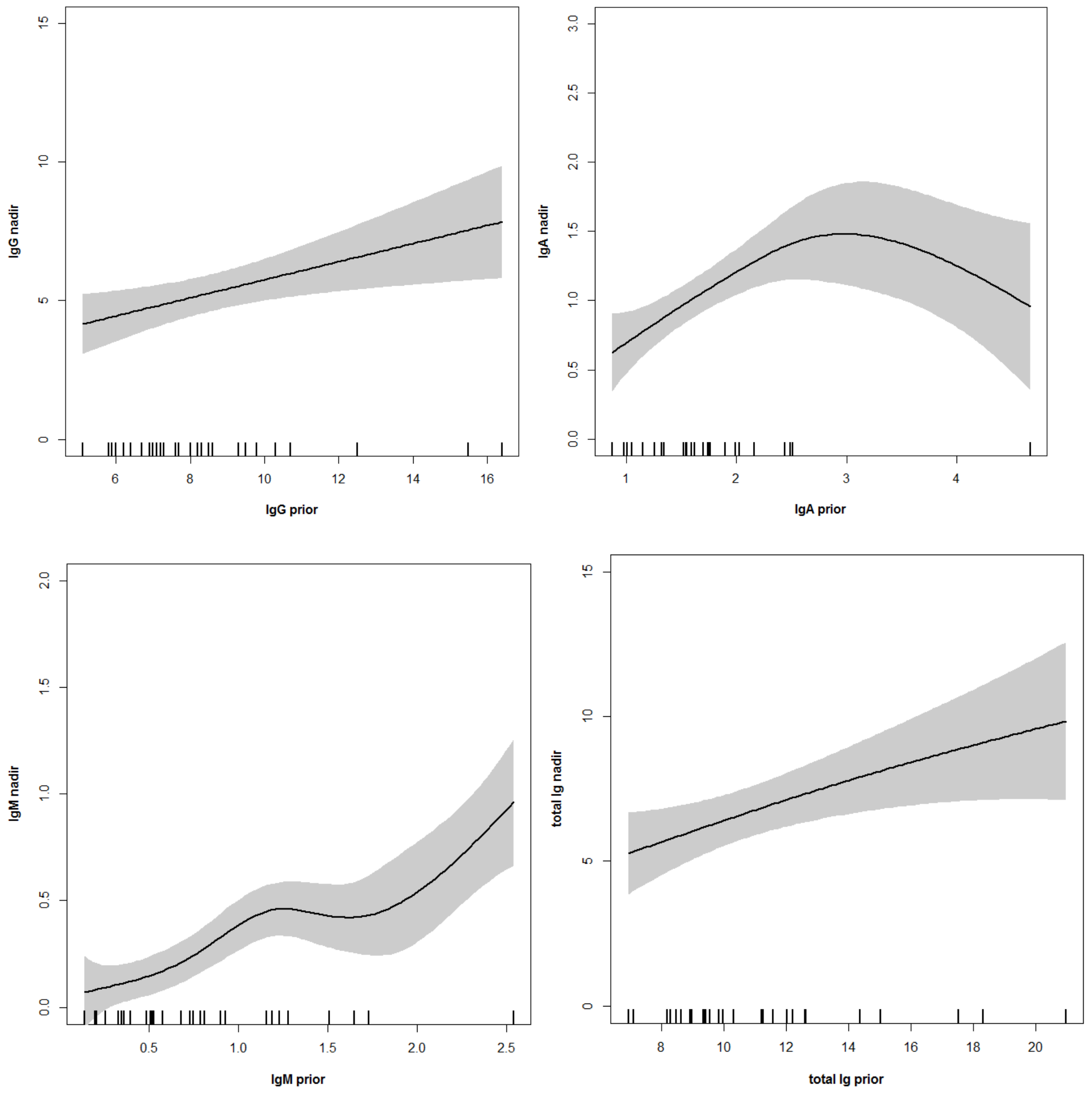

Figure S2

Two-dimensional biplot results using age and CYC cumulative dose with severe infection probability contours

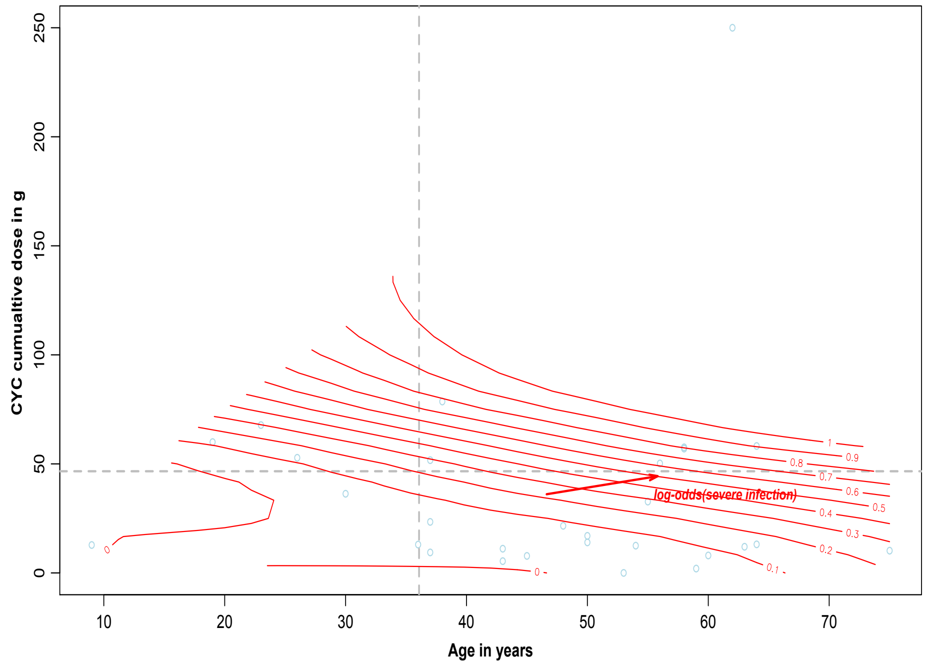

Figure S3

Two-dimensional biplot results using CYC cumulative dose and total Ig level prior RTX with hypogammaglobulinemia (defined as total Ig <6 g/L) probability contours.

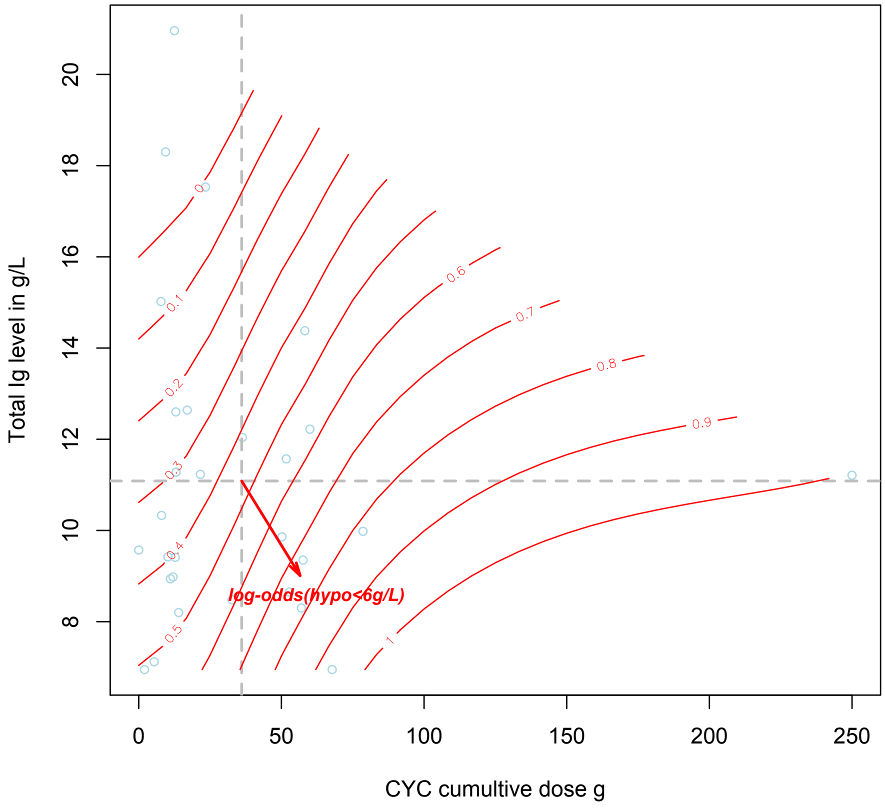

Figure S4

Two-dimensional biplot results using CYC cumulative dose and total Ig prior RTX with discontinuation due to hypogammaglobulinemia contours.

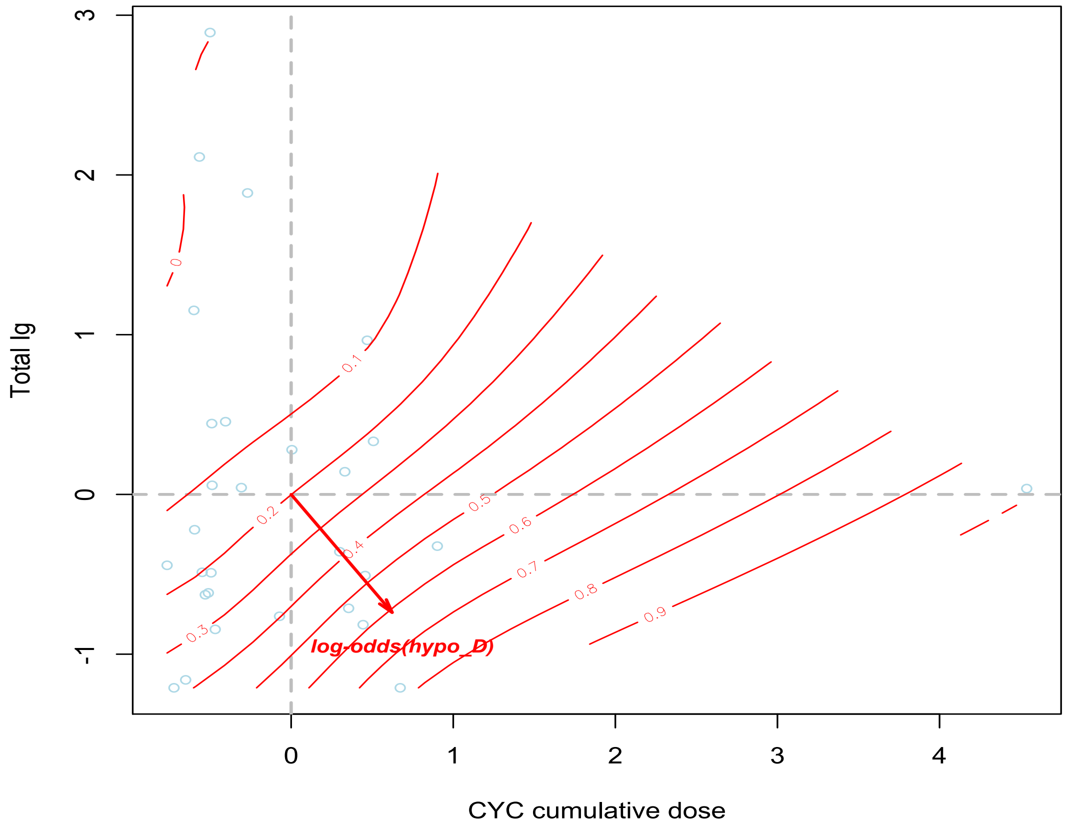

hypo_D: discontinuation of rituximab due to hypogammaglobulinemia
